# Supplementary material for: Patterns of Intron Gain and Loss in Fungi
Source: PLoS Biol. 2004 Nov 30;2(12):e422. doi: 10.1371/journal.pbio.0020422 (PMC532390; doi:10.1371/journal.pbio.0020422)
Supplement: Table S1 — Also available at http://genes.mit.edu/NielsenEtAl/. (4.3 MB ZIP). [file pbio.0020422.st001.zip › NielsenEtAl/html/631.html]

AN2717.1.NCU08435.1.MG06205.1.FG01582.1


```
 CLUSTAL W (1.82) Multiple Sequence Alignments - Introns Inserted


Sequence 1: NCU08435.1	1351 aa
Sequence 2: MG06205.1	1338 aa
Sequence 3: AN2717.1	1203 aa
Sequence 4: FG01582.1	1467 aa
Alignment Length: 1514 aa
Number Identitical Residues: 306 aa
Alignment Score (without introns) 16299


MG06205.1 	MEVFLSGLPTEQLTARSLEQQLTPLILKLGIGKHDFGCEKAARSKFANLTFRHPEQARLF
NCU08435.1	MEVYIRNLP-QGLSERSLTAHLKPFMDNLGIRHYD--CNKRSNQPFGKVIFITAREALNF
FG01582.1 	MEVICRNVP-LDLSEDNFKRELLPFMKALNISDFY--CEKPRRKSQAWIKFLSAQDGSAF
AN2717.1  	MEVFVHNLP-GNFTKDSLKHQLEPFMRSLAITDYD--CEKPRKKRFGHILFLNQSDGKRF
          	***   .:*   ::  .:  .* *::  * * ..   *:*  ..  . : *    :.  *

MG06205.1 	LRHYGEVPESSTNKISSLPQGQAQPSPFGRDRAPRSTARLRLLGKPIWCRKSIRAVNPIT
NCU08435.1	IAKHPRLYIMNQQAECRVSKPQP---------------------------KDLKNLVNGI
FG01582.1 	LARHGKQRRCENHGKENIDPSNPLNNFTG-----SHIFRSVPRDIARLHILKTAVFVEQS
AN2717.1  	LAHHGEERRAGRRRPLSKLNLLG-----------------------------SQVLCKPS
          	: :: .      .                                          .    

MG06205.1 	LRGMDESHRRHQEKE---SKALTG-HRDQDKHQHGVDFDVMGVACGHYAFVSQQ--LAYF
NCU08435.1	LYEAEEKERRKKAWE---ENTSNN-ARPARTPTLALDADVL--DCGHWSYIDGN--LTFI
FG01582.1 	TKPVDKYAIAHLKHEREKKKQSTTTSNNKGRHAPEKAFQVSAISCGGNTFHGPNEVITFV
AN2717.1  	HRKPDEFDLRALENE---VQERTNPSRIIEEEGGSVSFILSTASCGYTVFVDNE--FSYV
          	    ::        *    :  . . .            :    **   : . :  :::.

MG06205.1 	PEREWPWM---CKASFTKLNLHVRRSDGTIRMRIPLSTIHEIVWYTHDDRQGTLTLTLDS
NCU08435.1	TEWSSSLR---VSAKFAKHDLVIT-SLAESQVSIPYRYIHELVWSNG----GHVAVTLAH
FG01582.1 	DHHKLLFRPEAVTGKFTSQWLTIQ-VEHAHRMDFHNETIQDLIATRS---DMSITLVLTE
AN2717.1  	PEVELQQY---DSIIFRKRNITVH-LGNTPRIKIPLNTIYELVWSKH----GSLLLTLVT
          	 . .        .  * .  : :       :: :    * :::          : :.*  

MG06205.1 	VPWFLTEECTLDDLMARIAIGSGKIKENQQNRMQALDEQHAEIVGTCLTYHFNISSHSIS
NCU08435.1	APTFLSPPIPL--------LRPGQPK---RMRLEACDSNHAKVSKFCLVYHFKVSDKHLK
FG01582.1 	PPKMYTYRPPT---------FERSMQEWSRVHGLHAWGLPADCTLSCLVYRIYFYDT-IS
AN2717.1  	VPLFFQCDGHS------------------RIRLTSPGGPQAAVVGQCLVYQFQVVSSSSN
          	 * :                         : :        *     **.*:: . .   .

MG06205.1 	RDIKMLERKSFTNITR--YKLAIWNTPHPTLG---SFKDKALQLRQELKAATSASINSGK
NCU08435.1	QNQGSYRGSDFRTAINGLNEQEMFWVTHYTFAIQTTSPQDAYAVAVDRLRAALLEYEGRD
FG01582.1 	FDQVLVTLKNK-------NILAITNHDLPSVRWIEIGAESNHTIGRQTLVEKSKNLLTSG
AN2717.1  	LRARMGKVAEY--------DLSVINYEFKTVH-----NFDSWQSQLTLLRDRLGEYTRAG
          	         .            :      :.        .              .     

MG06205.1 	SLPHSHLFVLQALFYNGYLHPATVLEILHQLFRRFYEDRLSG--RTFIAPEAVKSLMDAE
NCU08435.1	TLPYSLLFNLQALVYMSYLHPTTVLKLAKRLADMFETARRSGQRQDPISVDAFKDLFKTI
FG01582.1 	RVHFALLFQVQALVWNNFINPASGIKLLDLLECVIEDHKKRK-VAVPVTTDAMKKLFQTI
AN2717.1  	TLPFGILFQLQALVYNGYLPPNIVLRLTRKLASITKTRKVQG--RRPISVNSMKRLFNLI
          	 : .. ** :***.: .:: *   :.:   *       :        :: ::.* *:.  

MG06205.1 	CIPWP--SPYEDPEYFEVSSLMKWIYEKNEEFQGG--FDLRTGQLRQNSNLTHIYRVVVT
NCU08435.1	DWPSPSLSSAEELAQFEVEGIIEHLKKTEKRMREG--YTLRLNEEIP-PGLTKIYRALVT
FG01582.1 	PYPCP----GTEPKELDVVSIMTNIMDAEYEARKDDPERDRIYGGKLPPNQIWVFKALVT
AN2717.1  	DWPSP----FGDPEEFSIKRLLEVITGNDKDLGEG--ASEAEALFQPAPHLAHIHRVVVT
          	  * *      :   :.:  ::  :   :     .             .    :.:.:**

MG06205.1 	PTRITLHGPEIENKNRVLRKYPKHQDCFVRVQFCDENTQDIRFSPKISYDRVYSRFLDVL
NCU08435.1	PTRIELHGPELEAKNRILRKFPEHQDHFLRVQFAEEDGQDLFFNSAVSMDAIYQRFKDVL
FG01582.1 	PTRLLLDGPEAESKNRVLRKYADYNDYFLRVLFCEEGGQDLRFHPKVDNEPIYSQFRRMM
AN2717.1  	PSRITLHGPETEPLNRILRRFPNHHEYFIRVQFCDENGEDIYFNPRINNSLIFARFKNVF
          	*:*: *.*** *  **:**::..::: *:** *.:*. :*: * . :. . :: :*  ::

MG06205.1 	NRGIQIAGRVYNFLGFSHSSLRTHSAW~FSAPFFDQ-GSQHTHASIIKSLGEFSSIQCPA
NCU08435.1	TNGISVGGRVYRFLGFSHSSLRAHSLW~LAAPFIYD-GKLQLASNIIEDLGDFRNIMSPA
FG01582.1 	DHGIQIAGRKFDFLGFSHSSLRSHSAW~FSAPFIDKDRQLQSPEAILQALGDFRDIRVPA
AN2717.1  	ANGFQIAGRTYTFLGFSHSSLRSRSAW0FSAPFIDDDGSLQSYFTIIKAIGRFSEITSPA
          	 .*:.:.** : **********::* * ::***: .. . :    *:: :* * .*  **

MG06205.1 	KCAARIGQAFSETPSVVDLRDHDVEVVTHFPDITNRDGSRVFSDGVGKISESMMHIIWEN
NCU08435.1	RRAARIGQAFSETPYSVSLYDHGIDVIRQR-DVKRNE--RVFSDGVGIISQGALEVIHRE
FG01582.1 	KCAARIGQAFSETPYAVPIFENNIVPRYIP-DVKSEDGSRVFSDGVGTISQEALEAIWPY
AN2717.1  	RCAARIGQAFSETPFAVNLKENGIRVLRIP-DVQVGG--RVFSDGVGTISAKAVDIIHGV
          	: ************  * : ::.:       *:      ******** **   :. *   

MG06205.1 	IPMKKNAPTCFQVRWGGAKGMLALDMTLKGTKVCFR-DSMTKFESDDVHQLEICDSGSKP
NCU08435.1	IPESKGYPNCLQVRWAGAKGMLALDARLTGRQICIR-DSMEKFRSRDEEHLEICDMASKP
FG01582.1 	LSMNSAAPTCLQIRWGGCKGMLSLDTRLEGKVFCIRQESMMKFKSEDTGELGICDTASKP
AN2717.1  	LPPRNGAPTCFQIRLGGAKGMLSLDPRLRGSKICIR-PSMTKFQTDDQVNVEICGSGSKP
          	:.  .  *.*:*:* .*.****:**  * *  .*:*  ** **.: *  .: **. .***

MG06205.1 	IPLVLNRQMIKILEDMGTDRSWFMRLQSYRLQELRSITASAYNTWHFLDAQNVGQAIKLG
NCU08435.1	IPLMLNRQMIKILEDMRAPAQWFLELQEKELQRLRAITDNVQNVATFLKLQCVGDSVHLS
FG01582.1 	LRLVLNRQMIKILEDMGTRDEWFFELQNNALDILRNVTADATNTSSFLEHQAIGINMGLP
AN2717.1  	IPLFLNRQVIKILEDMGVDREWFMTLQELRIEQLRKATATTKNTATFVKGQSVADCVKLY
          	: *.****:******* .  .**: **.  :: **  *  . *.  *:. * :.  : * 

MG06205.1 	KFINFADKHGMDYRTDDFMRSVVEAVVLRELRLLKHKARIPVKYGKTLFGIIDETGYLRE
NCU08435.1	QFLKDLDKMNIDYRRDQFLRGIVEAVVLRELRLLKHKARIPVPYGVTLFGVMDETGLLRE
FG01582.1 	RLVKQLEKMDIDYRRDKFLKAAVEHVVLRELRLLKHKARIPVDNGVTLFGIMDETGFLKE
AN2717.1  	RLFLLFHNLKLDYKKIPFLRAVVEAMVLRELRLLKHKARIPVHKGITLYGVMDETGFLNE
          	::.   .:  :**:   *::. ** :****************  * **:*::**** *.*

MG06205.1 	GEVVISFGFSQTGEDQ--PTPPQDGQIVIVTRSPALHPGDIQIAKQVVPPPSHPLSHHIN
NCU08435.1	GEVYVTF---ETVDGR--FKDPPTAGPVVVTRSPALHPGDIQIAHNAIPPAGHPLRELKN
FG01582.1 	GEVYVTYDKTYGRNNSRGIKSTLTDGEIIVTRSPALHPGDIQLVKQVTPPQGHSLRSLQN
AN2717.1  	NEVFVTY----QAMDR--LAAPPQNCRLLVTRSPALHNGDIQYPYNVVPPKGHPLQQQRN
          	.** :::       .      .     ::******** ****   :. ** .*.*    *

MG06205.1 	CIIFSQHGQRDLPSQLSGGDLDGDIYNVIWDGDAMP-KETFDPADYPRVEAQDIGRRVVS
NCU08435.1	CIVFSQNGERDLPSQLSGGDLDGDTFNVIWDQSIVATLRTFAAADYPRVEPLKLNREVES
FG01582.1 	CIVFSQKGKRDLPSMLSGGDLDGDIYNIIWDPEAMP-SRVFRPADYPRVTPQPLDRQVEP
AN2717.1  	CIVFSQKGTRDLPSQLSGGDLDGDLYNIIWDPAAAP-KRVFTPANYPRNPPLDIKRVVER
          	**:***:* ***** ********* :*:***    .  ..* .*:***  .  : * *  

MG06205.1 	ADMAQFFIDFMKTDHLGVIATRHVILADQLDDGTLSQECRTLAGLHSTAVDFSKSGIPAE
NCU08435.1	KDMADFFVEFMKADHLGVIAVRHMILADERNEGTLDADCLKLAALHSKAVDFSKSGIHVD
FG01582.1 	KDIADFFINFMRTDILGVIATRHVILADYNDLGSIDRDCIALAELHSTAVDFSKTGIPVN
AN2717.1  	EDMANFFVEFMQSDRLGVIATKHMILADQMELGTSHSDCKKLAQLHSTAVDFSKTGIPVR
          	 *:*:**::**::* *****.:*:****  : *:   :*  ** ***.******:** . 

MG06205.1 	LKALPRANKFRPDF2LSPGPITNYHDKGKITLEEYTISEEAEEEEEEGDGPRYGYYRSEK
NCU08435.1	ITELPRPPMYRPDF2LVNGPDIKIHDKSTIDMEEQYLRQDDDDGD---DTPRYKYYKSDK
FG01582.1 	VKALPRAPRFRPDF2LATAPPLKLYDKGQLAHIGEDDEMDDADDMAR---TKTNYYQSEK
AN2717.1  	LKEMPFVNRSRPDF2LAPGPLAKIHDRQNIQLEARYVHPYPDDDID--FSPVHKYYRSEK
          	:. :*     **** *  .*  : :*:  :            :       .   **:*:*

MG06205.1 	VLGQLYREIDEEKIWNENIRVAVDHNKPSFWETFIDWAIKSAATVGA-VSWPVRIDSARR
NCU08435.1	ILGRLFRAVDEKKIWTKNIKLEVPSGGVPFWKEVESSLLKRVRGIGQ-VQWQHRLDEARR
FG01582.1 	ILGQLYRNVDEKKIWDEDIHRRINTAGPSVWDQLLTIVENEAIKYKLDIDWKRQSQEAWK
AN2717.1  	ILGKLYRAINEHEIWTKDIHTTVNSNPTAVWKRIIDSLTGRCIALGAPVNWKAHVETARQ
          	:**:*:* ::*.:** ::*:  :     ..*. .              :.*  : : * :

MG06205.1 	LK------SA~YDDHVRGHMSKYSDHPVNPISEREVFIGAILNKTGVQTHRQRDASHKLR
NCU08435.1	IC------ES2YEDGIKDAMVEFADSPTQPLKELEVVMGFILNKKGIQSRRQRDKSSKLS
FG01582.1 	IR------GL2YESSIEDKRWHFSENPRVPLTEVEVFCGFILNKRGAQTRRQGDASIKLK
AN2717.1  	LRSLYYILPT2YEDSMSNAMANFSKHPIYPLTELEVFIGQVLNNSGAQTNRQRERSIKLN
          	: :        *:. : .   .::. *  *:.* **. * :**: * *:.** : * ** 

MG06205.1 	DEFERTANHFARQMRMGNEGPDCVPLTGYMAELGPSAALDLCLACVCVASEQ-DVPLVAS
NCU08435.1	DAFARITKMVTNVLRP------STPPEEATSELH---ALELCLACFYVAGEKKSQPQESW
FG01582.1 	EEIDRTMTWIVKQIRDRGVGDGAETLSTVTEADGTTSRWREDVVELCWACVAVSCIKKDN
AN2717.1  	DEFERIASFILSQMRP--------------------------------------------
          	: : *  . .   :*                                             

MG06205.1 	RLGISK-----SPSR~RAAGRNGRKDGENSELQS-FRLVAATQFLRELRVKERELGVGEA
NCU08435.1	KRQIATGL---ESFR~LVAGSALLLEIKAQEQKIRLRHAARSGGFVGVRGGSRVAGRGGR
FG01582.1 	APELYHGNGELESFH~IVAASCLVRELNN--LARKMETAAGGGFVGVGCDGRSGRGGRGG
AN2717.1  	-SGLITG-------Y0VVSACALLMEVEELEKSRRLYGENRRPLLDAFTSSRKEKNDYE-
          	   :  .          .:.     : :  .    :        .          .    

MG06205.1 	GG~K---------------------GGGGF0QLQI~GPALQVGK~VVNS-SVWWLCKG--
NCU08435.1	GG~HRQPARVDVNTAAEAATLQTADTGAAF~GLGS~PSSTSAPT~TPSSGSNYTPSSGSD
FG01582.1 	KN1GIECCLMEIKDMDMDTGMSTEGLCASD~DLRQ0ERALNARI2HGGSDSVECVSEEHR
AN2717.1  	--~---------------------------~----~PKGLDRAT~QKTT-----------
          	                                      . .       :           

MG06205.1 	--MGIFGS------------------------------HEFGGKRYPEDVGLKYSKAGEL
NCU08435.1	MRDGIFNSPTG-----------------PYAGYQPELVHAQAGARTAGTSGGQVPSAAPA
FG01582.1 	LDFAQTNTWIVNSKLPDSLAKTIEHQKNKNQFPNRVDPYPSAVPWFSEEDRNRLSNKALL
AN2717.1  	--------------------------------------FEYGERWTAADVEAMLRQLELN
          	                                      .  .    .        .    

MG06205.1 	H-----------------------------------------------------------
NCU08435.1	NMPVRLAPADKVAH-------------------------LAALYAQMQIYQRH-------
FG01582.1 	AISAAPRDSNTGVHGRANQLKKIRVPGMRNMTINRDLWQVKASYTRMASLKRNAFTPLLL
AN2717.1  	TIV---------------------------------------------------------
          	                                                            

MG06205.1 	----------------------
NCU08435.1	----------------------
FG01582.1 	VATMMTISDKLSAWKCLRYVGI
AN2717.1  	----------------------
          	
```
